# An Appeal for a Change of Tactics

**Published:** 1911-02-11

**Authors:** William J. Robinson

**Affiliations:** New York, President of the American Society of Medical Sociology.


### AN APPEAL FOR A CHANGE OF TACTICS.

By WILLIAM J. ROBINSON, M.D. New York, President of the American Society of Medical Sociology.

He who is not a frequent visitor to Radical clubs, who does not come in contact with newspaper men, and who does not read regularly the numerous naturopathic, health, and physical culture journals, and other allegedly advanced publications, can have no idea how the medical profession is ridiculed, how it is maligned, how it is lied about, how it is misrepresented, how it is " knocked " on every possible occasion.

We are pictured as ignoramuses, " grafters," butchers, anxious to operate whether there is a necessity or not, drug dopers, " out for fees," etc., etc. We are denounced as a trust, a monopoly, a trade union, and any attempt of ours to organise, to pass laws protecting the public health, is characterised as an attempt at class legislation, a desire for special privileges, inspired by our fear of competition, by our fear of the superior skill of our irregular rivals.

The average physician, who has not given the matter any thought, has no idea what effect these unceasing slanders and persistent lies have on the public mind, how suspiciously a large part of the public is beginning to look at the medical profession, how we are losing the confidence of the people, how the ground is slipping from under our feet. As an illustration we need only mention the reception that has been accorded to the suggestion of a Federal Department of Health in America. The motives that actuate us in the States and the objects of such a department were at once misrepresented; the public was made to believe that its freedom to choose a medical adviser was threatened; the forces of reaction and obscurantism, masquerading in some instances under the guise of liberalism, were quickly marshalled, and in a short time a society was organised to oppose the scheme, which now claims a membership of one hundred and fifty thousand.

We, members of the medical profession, are ourselves to blame for this misunderstanding and ignorance. When the irregular fantastic and pernicious cults began to make their appearance we paid no attention to them. We thought they amounted to nothing and would soon dry up and shrivel away of themselves. When malicious attacks began to appear in the various quack publications we remained silent. We considered it *infra dignitatem* to pay attention to them, and we thought that the public would have no difficulty in seeing through their falsity and meretriciousness. Our long and patient inactivity has been due to the false idea that the truth will always triumph, and error is bound to die. Yes, eventually. But if error is allowed to grow and spread unhampered, while those who see the truth will not take the trouble to proclaim it and expose the error, then it can take centuries before the correctness of the truth and the falsity of the error will be perceived.

In this, as in every other line of human activity, prevention is immeasurably superior to cure, and the right way to fight is not to permit it to get a firm foothold. Error and superstition are hard things to uproot after they have attained the dignity of a universal belief.

It is time that the medical profession changed its tactics and assumed a wideawake, militant attitude. It is time that we actively attack error wherever it shows its head. By reading papers before lay audiences, by participating in discussions, by writing to the newspapers, by refuting the false arguments of false prophets wherever they appear, we can do much towards destroying the influence of the quacks and the irregular cults. In short, we must throw off our exclusiveness, we must go out to the public and take it into our confidence.

The truth is with us—that we know; only we must not hide it under a bushel and expect that its light will, without any effort on our part, penetrate into the darkest recesses of ignorance and quackery.

